# Rapid progress on the vertebrate tree of life

**DOI:** 10.1186/1741-7007-8-19

**Published:** 2010-03-08

**Authors:** Robert C Thomson, H Bradley Shaffer

**Affiliations:** 1 Department of Evolution and Ecology and Center for Population Biology, University of California, Davis, CA 95616, USA

## Abstract

**Background:**

Among the greatest challenges for biology in the 21st century is inference of the tree of life. Interest in, and progress toward, this goal has increased dramatically with the growing availability of molecular sequence data. However, we have very little sense, for any major clade, of how much progress has been made in resolving a full tree of life and the scope of work that remains. A series of challenges stand in the way of completing this task but, at the most basic level, progress is limited by data: a limited fraction of the world's biodiversity has been incorporated into a phylogenetic analysis. More troubling is our poor understanding of what fraction of the tree of life is understood and how quickly research is adding to this knowledge. Here we measure the rate of progress on the tree of life for one clade of particular research interest, the vertebrates.

**Results:**

Using an automated phylogenetic approach, we analyse all available molecular data for a large sample of vertebrate diversity, comprising nearly 12,000 species and 210,000 sequences. Our results indicate that progress has been rapid, increasing polynomially during the age of molecular systematics. It is also skewed, with birds and mammals receiving the most attention and marine organisms accumulating far fewer data and a slower rate of increase in phylogenetic resolution than terrestrial taxa. We analyse the contributors to this phylogenetic progress and make recommendations for future work.

**Conclusions:**

Our analyses suggest that a large majority of the vertebrate tree of life will: (1) be resolved within the next few decades; (2) identify specific data collection strategies that may help to spur future progress; and (3) identify branches of the vertebrate tree of life in need of increased research effort.

## Background

**Figure 1:**
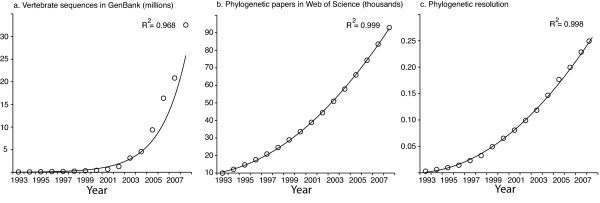
**Cumulative phylogenetic information amassed for the last 16 years**. The accumulation of sequences for vertebrates in GenBank (a), papers using the term 'phylogeny' or 'phylogenetics' in the Web of Science database (b) and phylogenetic resolution (measured as the proportion of nodes with at least 50% bootstrap support) in the vertebrate tree of life resulting from these research efforts (c). In all cases, the data are cumulative from the start of each analysis. Phylogenetic resolution is calculated as in Table 1. Trend lines are exponential in (a), and second order polynomial in (b) and (c).

Resolution of a well-resolved phylogeny for all species is a central goal for biology in the 21st century. Inference of this 'tree of life' has far-reaching implications for nearly all fields of biology, from human health to conservation [1]. As efforts have shifted from primarily morphological to molecular approaches, a number of complex methodological issues central to the reconstruction of large phylogenies containing hundreds to thousands of species have been identified and, in some cases, solved [2-4]. At the most basic level, however, progress on the tree of life is limited by data. Both the rates at which DNA sequences are gathered and species are sampled have increased at a dramatic pace, leading to the now well-known exponential accumulation of basepairs in GenBank (Figure 1a) [5]. At the same time, the number of studies that infer and/or apply phylogenies has also grown rapidly (Figure 1b) [6]. While these indications of progress on the tree of life are encouraging, they are indirect and fall short of quantifying the growth of phylogenetic knowledge.

**Table 1:** Species sampling and phylogenetic resolution for major vertebrate clades

Group	Species diversity	Proportion of species with data	Resolution (50% BP)	Resolution (95% BP)
Ray-finned fish	29737	0.26	0.14	0.05
Amphibians	6420	0.45	0.30	0.13
Birds	9953	0.63	0.39	0.14
Cartilaginous fish	1158	0.45	0.19	0.04
Crocodilia	23	1.00	0.80	0.60
Mammals	5488	0.62	0.40	0.15
Lamprey	41	0.61	0.32	0.08
Squamates	8396	0.45	0.27	0.09
Turtles	321	0.77	0.59	0.26
All clades	61259	0.41	0.25	0.09

Values were calculated by combining results from each of the 100 clades into nine larger groupings. 'Proportion of species with data' is calculated as the number of species in each group represented by at least one sequence in our dataset, divided by the total number of species described for that group. Resolution is calculated as the number of nodes resolved for each group divided by the number of nodes in a fully bifurcating version of the same tree for 50% and 95% bootstrap (BP) majority rule consensus trees, respectively.

GenBank is composed of sequences stemming from a variety of interrelated disciplines (for example, systematics, population genetics, and genomics). When combined (as in Figure 1a), these sequences form an enormously heterogeneous pool of data, much of which is not directly informative about phylogeny (for example, genome re-sequencing projects). Likewise, many of the publications summarized in Figure 1b employ previously proposed phylogenies, or use existing data in different ways, and may not represent new information about the tree of life. As a discipline, phylogenetics lacks a direct measure of the rate of progress on the tree of life and the overall difficulty and scale of the problem of inferring the tree of life is therefore poorly characterized.

Given the massive research effort that has, and will be, allocated toward resolving the tree of life, an understanding of the scale of the problem is important. It appears that the pace of progress is accelerating as methods for phylogenetic inference mature and data become easier to collect. Inferring the rate of this progress, however, is not straightforward, though the interest in doing so is widespread [7,8]. Previous work examining the phylogenetic signal present in large sequence databases suggests that these resources contain a wealth of phylogenetic information [9,10]. As a result of the well-established practice of depositing molecular sequences in GenBank upon publication, this database probably represents the single biggest repository of phylogenetic data in the world, making it the most important repositories for information about progress on the tree of life. Like any large-scale resource, the data contained in GenBank are heterogeneous in terms of quality of annotation information, sequence lengths, taxonomy and other key issues, which makes combining and utilizing these data on a large scale a major challenge. However, given the breadth of GenBank, and the longevity of the database (it is now nearly 20 years old), it also represents a unique resource for tracking phylogenetic progress.

Here, we measure progress on the tree of life using GenBank data for one particularly well-studied clade, the vertebrates. Vertebrata contains over 60,000 described species and is among the most well-studied segments of phylogenetic diversity [11]. The deeper portions of the vertebrate tree are becoming reasonably well understood [12-19] and many of the remaining problems are nearer the tips of the tree, at the family, genus and species levels. We, therefore, developed an automated supermatrix procedure to infer phylogenies for a large sample of vertebrate diversity targeted at these shallow levels of divergence (see Methods section and Additional File 1). We applied our supermatrix approach to track yearly progress since 1993--the year that most data deposition in GenBank began--and document a rapid, but skewed, rate of phylogenetic resolution across vertebrates.

By focusing on the annual additions to the database, we are able to measure the past rates of phylogenetic progress and generate predictions for the completion of a species-level vertebrate phylogeny. Further, we examine phylogenetic efforts to date, in terms of taxon and gene sampling, and make recommendations for increasing the effectiveness of future efforts. Our complete dataset includes 227,329 sequences from GenBank's core nucleotide database (release 167.0) for 100 vertebrate clades, which encompass a total of 29,237 described species. Of these species, 11,996 have at least one sequence deposited in GenBank and so were included in our analyses (Table 1, Additional File 2). Using these newly estimated phylogenies, we calculate two simple metrics of phylogenetic resolution based on the fraction of the nodes that are resolved at the 50% and 95% bootstrap support levels for each of the 100 clades (see Methods section). By reconstructing data availability, and estimating trees and resolution metrics for each clade over GenBank's history, we track the accumulation of vertebrate phylogenetic information through time.

## Progress on the vertebrate tree of life

### Major trends

Our analyses indicate that progress on the vertebrate tree of life has been remarkably rapid over the last 16 years, resulting in an at least 50% bootstrap support for approximately one quarter of the nodes in the vertebrate tree of life (Figure 1c). The increase in resolution has been faster than linear (linear *r*^2 ^= 0.970; second-order polynomial *r*^2 ^= 0.998; *P*-value for significant increase in *r*^2 ^= 4.13 × 10^-12^) and appears to be proportional to the increase in number of publications on phylogenetics.

**Figure 2:**
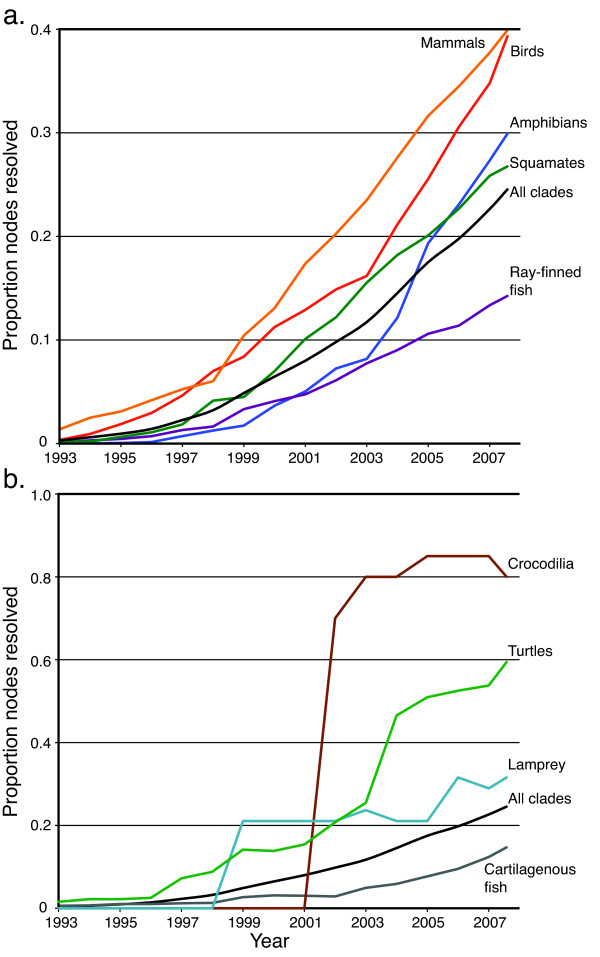
**Phylogenetic progress among major vertebrate lineages**. Phylogenetic resolution as a proportion of the total possible nodes resolved (measured as the proportion of nodes with at least 50% bootstrap support) in high (a) and low-diversity (b) major vertebrate lineages. The black line in each panel shows the overall proportion of nodes resolved in vertebrata, calculated for all species pooled; note the different scale of the y axis in panels (a) and (b). The small decreases in resolution for the Crocodilia and lamprey are due to stochastic effects and the small size of the clades (see Additional File 1).

**Figure 3:**
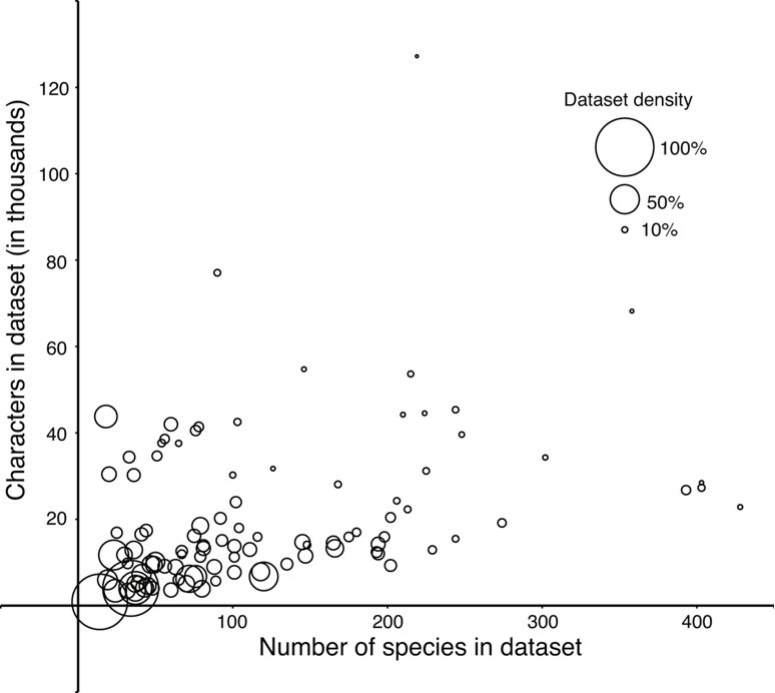
**Scatterplot of dataset characteristics**. Each circle represents the characteristics of the complete 2008 data matrix from each of 100 vertebrate clades. The number of species in the dataset (*x*-axis) refers to the number of species sampled for the clade, not the total described diversity of that clade. The number of character refers to the number of columns (nucleotides) for that matrix, rather than the total number of sequences sampled. The size of each circle represents the density of each dataset measured as the percentage of non-missing data.

When the 100 clades are pooled into major vertebrate lineages (classes or similar taxonomic levels), several important trends emerge (Figure 2). Among high-diversity clades (Figure 2a) phylogenetic progress has been most rapid for tetrapods and, in particular, for mammals and birds. However, progress has been even more striking for relatively low-diversity clades (those containing < 2% of vertebrate diversity) such as crocodilians and turtles (Figure 2b). Crocodilia, with only 23 contained species, is particularly well resolved. We recovered 80% of the nodes in its tree (Figure 2b), 60% of which were well supported at a bootstrap level of 95 (Table 1). This trend is general across the clades that we sampled. Large clades, on average, experience less research effort per contained species than small clades, resulting in data sets with large amounts of missing data (Figure 3) and comparatively low resolution (Figure 2a and 2b). Among the high-diversity vertebrate lineages, we found three relatively distinct levels of progress. Birds and mammals have undergone the most rapid phylogenetic progress, with approximately 40% of each group's tree of life resolved (Figure 2a), followed by amphibians and squamate reptiles (~30%) and ray-finned fishes (~15%). Our estimate of 40% resolution of the mammal tree of life is similar to a recent species-level analysis of most mammal species [12], suggesting that our automated supermatrix approach is performing reasonably well. The study by Bininda-Emonds *et al*. [12] found a 46% resolved supertree for mammalia, but included all the deep-level nodes in the mammal tree which tend to be better resolved than the tip level nodes and, thus, increase the overall resolution.

Another major trend in the accumulation of vertebrate phylogenetic knowledge is that marine clades are the least well characterized of all major vertebrate lineages. Ray-finned fishes are extremely diverse, containing over half of all vertebrate species, and this may explain their low (~14%) proportion of resolved nodes. However, the cartilaginous fishes (sharks, rays and skates) and lampreys are both species-poor (with ~1200 and ~40 species, respectively) and have a similarly low resolution (Figure 2b), suggesting that phylogenetic progress on marine 'fishes' has lagged behind the remaining vertebrates, regardless of species diversity *per se*. The cetaceans (whales, dolphins and porpoises) represent the only exception to this trend in our dataset, with 100% species-level sampling and 61% of nodes in their tree of life resolved (Additional Files 2 and 3).

Although progress has increased steadily for virtually all clades, it has improved dramatically for a few groups. Phylogenetic resolution in the amphibians was accumulating at the same slow rate as ray-finned fishes throughout the early period of molecular systematics, but then saw the most rapid phylogenetic progress of any diverse clade beginning in about 2003 (Figure 2a). This increase was probably due to a large influx of funding and several prominent studies on amphibian systematics in the last several years [16,20-25]. Both amphibian and bird research received major National Science Foundation funding from the Assembling the Tree of Life initiative (in 2004 for the amphibians and in 2002 and 2003 for birds [26]), which was immediately followed by rapid increases in phylogenetic progress.

### What dataset features are associated with phylogenetic resolution?

As vertebrate phylogenetics moves forward, we can ask what gene- and taxon-sampling features are most strongly associated with phylogenetic resolution: such insights should aid the community in allocating resources for future research. For example, among the 100 clades that we analysed, species sampling in GenBank varied between 6% (for the *Paracanthopterygii*) and 100% (for Cetacea, Crocodilia, Lemuriformes and Perrisodactyla) of described species (Additional File 2). Regardless of the measure (gene, taxon or character sampling), the amount of effort has been uneven across clades (Figure 3, Table 1, Additional File 2), resulting in a correspondingly uneven distribution of phylogenetic information (Figure 2, Table 1, Additional File 2). In order to assess the effects of sampling patterns on phylogenetic resolution, we performed a multiple regression of the proportion of species sampled in a clade, the total number of species in the clade, the average number of characters per species and dataset density (or proportion of non-missing data) on phylogenetic resolution for the 100 clades (*r*^2 ^= 0.93, *P *= 9.3 × 10^-55^). The proportion of species sampled in a clade has the greatest effect on phylogenetic resolution (partial regression coefficient 0.80), followed by dataset density (partial regression coefficient of 0.22). Both clade size and number of characters have smaller, but significant, impacts (partial regression coefficients of 0.10 and 0.11, respectively). Dataset density co-varies with clade size and number of characters (Figure 3), with exceptionally large clades having both fewer characters as well as lower dataset densities.

### Are we concentrating on the best genes?

While confidence in the tree of life will ultimately come from rigorously analysed multiple marker datasets, the first approximations will most likely come from sparsely sampled (at the gene- and character-level), taxonomically enriched datasets. Following the large effect of taxon sampling, the density of these matrices appears to have the largest effect on the resolution of the resulting trees. To this end, researchers can help to increase the data density in the 'vertebrate matrix' by focusing on a common set of markers, in addition to clade-specific markers. Ideally, this common set of markers should comprise the most informative and the most commonly used markers.

We examined sampling efforts by identifying those genes that have been most heavily sampled for phylogenetic studies and asking whether those genes also carry a strong phylogenetic signal. We selected the most heavily studied subset of the 100 clades (defined as clades that had been sampled for more than 20 genes, *n *= 37), analysed each of the genes for these 37 clades independently and asked which genes had received the most sequencing effort (measured by the number of taxa that had been sequenced) and which genes provided the most resolution (measured by the relative amounts of resolution found in phylogenies derived from each gene). The most frequently sampled genes were (in decreasing order) the mitochondrial markers cytochrome B, 12 S and 16 S ribosomal RNAs. These mitochondrial genes were among the top five genes in terms of taxon sampling in 89%, 76% and 73% of the clades, respectively. The most frequently sampled nuclear genes were recombination activating gene 1 (RAG-1), β-fibrinogen and myoglobin, which were among the top five gene clusters in terms of taxon sampling in 22%, 19%, and 16% of the clades, respectively.

The most highly resolved gene trees in our dataset were derived from the mitochondrial nicotinamide adenine dinucleotide dehydrogenase subunit 2 and control region, the nuclear β-fibrinogen, aldolase B, myoglobin, growth hormone 1 and RAG-1. Thus, among the nuclear genes, the most heavily sampled genes were also among the most resolved genes, although this analysis also identifies growth hormone 1 and aldolase B as strong candidates for the additional sequencing effort. In the mitochondrial data, the genes with the most resolving power were not among the most heavily sampled. Further, the most heavily sampled mitochondrial genes did not rank near the top of mitochondrial genes in terms of resolving power. Cytochrome b, 12 S and 16 S ribosomal RNAs, rank at numbers 8, 9 and 10 (out of 16) in terms of the resolving power for mitochondrial genes. Despite these results, an attractive target for additional mitochondrial sequencing in vertebrate phylogenetics is, perhaps, cytochrome oxidase I. This gene is already the target of massive DNA bar-coding efforts and ranked well in our analysis, in terms of both species sampling (four out of 16) and phylogenetic resolution (five out of 16).

We checked that these results were not being driven by a correlation between the number of species sampled for a gene and phylogenetic resolution and found no significant relationship (*r*^2 ^= 0.0015, regression slope = 9.7 × 10^6^, *P *= 0.15). The analysis is based on averages across several clades and it is well known that rates of molecular variation vary across the tree of life. Further, these recommendations apply to studies at relatively shallow levels of diversity (generally at the family level and below). Thus, these genes appear to be attractive starting points for a common gene set, although it is unlikely that they will be the most informative genes in all clades and certainly not at all phylogenetic scales.

### Future progress on the tree of life

**Figure 4:**
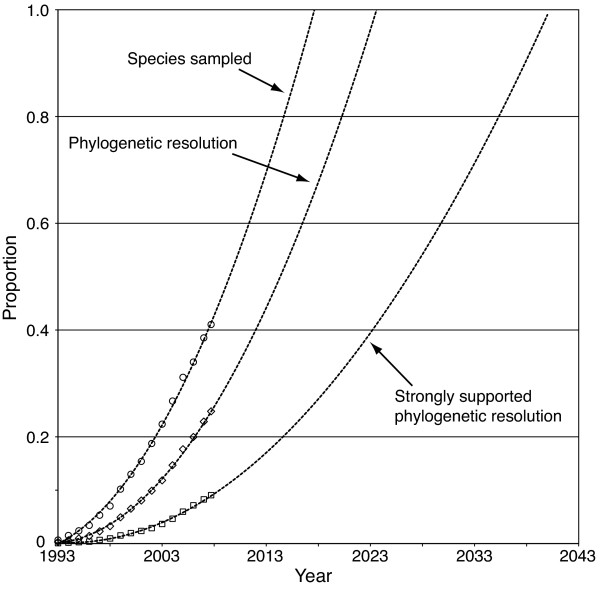
**Projections of future progress**. Projection of the best-fitting trend line for species sampling, phylogenetic resolution and strongly supported phylogenetic resolution. Phylogenetic resolution refers to the proportion of nodes resolved in a 50% majority rule bootstrap consensus tree. Strong support refers to this same measure in a 95% majority rule bootstrap consensus tree.

Extrapolations based on the last 16 years provide a framework for the discussion of the future progress on the vertebrate tree of life. Like any extrapolation, these projections are assumption-laden and are necessarily approximate, although they are instructive. If current trends continue, we predict essentially complete species-level sampling before 2020 (Figure 4). This assumes that all species are equally easy to sample and that no new vertebrate species will be described, both of which are incorrect assumptions. Presumably the last few species of many clades will be those that are rare and/or secretive, subject to political difficulties with collecting the data or are recently extinct. Even so, current trends predict that most species will have sequenced DNA available in roughly another decade. Although a single sequence for a single specimen is a far cry from a complete phylogeny, our projections suggest that we will have at least a rough phylogenetic placement, based on molecular data, for most vertebrates in the near future.

Although projections for the resolution of the tree itself imply slower progress, they still suggest that an essentially fully resolved vertebrate tree of life is within reach. Again, these projections are based on strong assumptions, as some parts of the tree will be more difficult to resolve than others (for example, those with short branches, incongruous gene trees, etc.). If much of the tree is difficult, and our progress to date is largely made up of the 'easy' parts, future progress will take much longer than our projections. Alternatively, if we assume that our progress to date has been an unbiased sample with respect to easy versus difficult nodes, then the current rate of progress suggests that we will understand a majority of the vertebrate tree, with strong support, in the next three decades (Figure 4).

## Conclusions

Progress on the vertebrate tree of life has been surprisingly rapid, increasing polynomially since the early 1990s, when molecular phylogenetic approaches became widely used. Our analysis suggests that approximately a quarter of the nodes in the vertebrate tree of life are resolved with at least a moderate level of statistical support. While we expect the trends in Figure 4 to become sigmoidal over time, it appears that a substantial fraction of the vertebrate tree could be understood to a first approximation in the next few decades. Given the modest progress in the first few years of molecular phylogenetic work, this recent rise in phylogenetic progress is remarkable. The informatic pipeline that we use here can be applied to all clades represented in GenBank and such analyses should help determine the groups that are in greatest need of future phylogenetic research and, potentially, the genes that may lead most efficiently to their resolution. As this occurs, we look forward to the more comprehensive explorations of the patterns, processes and, perhaps most importantly, strategies for conservation that these phylogenies promise [27].

## Methods

### Data

We selected 100 non-overlapping clades for our analysis. In order to do so, we chose a species at random from the National Center for Biotechnology Information (NCBI) vertebrate taxonomy and then, for each species, chose the largest clade that included that species but had fewer than 500 species in the NCBI taxonomy. This is different from the total number of species in a given clade because the NCBI taxonomy contains only those species that actually have a sequence deposited in GenBank. For example, if we randomly chose the snapping turtle, *Chelydra serpentina*, the largest containing clade would be all turtles (310 species in the NCBI database), since the next most inclusive clade (Sauropsida) has 11,391, which is over 500 sampled species. The cut-off of 500 sampled species per clade was used in order to keep the datasets to a size that would allow tractable analysis times and memory usage, as well as maintain the molecular divergence present in the alignments at a reasonable level. For each of these clades, we downloaded all sequences in GenBank's nucleotide core database (release 167.0) that were between 100 and 5000 basepairs in length. This excluded very short sequences that were unlikely to be phylogenetically informative and were difficult to align, as well as extremely large sequences that require excessive memory in our automated pipeline. We also sought to exclude model organisms, which we defined as species for which greater than 10,000 sequences existed in the nucleotide core database, because most of the data available for these organisms is not phylogenetically informative for the scope of this analysis. Finally, we downloaded a list of the publication dates for all sequences, as well as the taxonomy file for each clade to use in downstream parts of the analysis.

We filtered sequences to exclude data that are unsuitable for phylogenetic analysis (for example, microsatellites, paralogs, repetitive elements) and standardized all taxon names contained in the deflines to the NCBI taxonomy to correct misspellings and standardize alternative taxon names. Finally, we excluded sequences that were not unambiguously assignable to a single species (for example, hybrids).

### Clustering, alignment, and matrix assembly

We assembled the sequence data for each clade into gene clusters by sorting the sequences with all-against-all BLAST clustering using BLASTCLUST (settings: -L 0.25 -S 75 -b T -p F -e 10E-5 -S 1). In order to assemble the yearly datasets, we pruned each cluster to include only those sequences deposited in GenBank in 1993 or earlier, 1994 or earlier and so on through 2008; this resulted in 16 sets of sequence clusters. We combined each set into a supermatrix using an automated pipeline based on an extension of the methods developed in reference [28] and containing the following basic steps: We removed duplicate sequences within clusters (that is, multiple sequences for a species from the same gene) keeping only the longest sequence for each species. We then aligned the sequences in each cluster using the local alignment algorithm implemented in DIALIGN [29], followed by the refinement algorithm from MUSCLE [30]. Next we identified the set of all clusters that were both potentially phylogenetically informative (contained at least four species) and overlapped with at least one other cluster in the set by four or more species and assembled them into a supermatrix (following [31]). This process yielded a total of 1192 supermatrices. Fewer than the 1600 (16 years × 100 clades) total possible supermatrices were constructed because no data were present in GenBank for several clades during the early years of data deposition in GenBank.

### Supermatrix analysis and rogue taxa

We conducted preliminary bootstrapping analyses on each dataset with PAUP*4.0b10 in order to identify rogue taxa, which are known to be particularly problematic for supermatrix analyses [28,31,32]. These should be distinguished from taxa that are phylogenetically unstable because of true ambiguity in the data (due to a paucity of informative characters, mutational saturation and so on), though these two causes of phylogenetic instability are not mutually exclusive. Allowing these rogue taxa to remain in the analysis can have extremely detrimental impacts on the resulting consensus trees and so their identification and removal is essential [28]. For these preliminary analyses, we carried out 100 parsimony bootstrap replicates using a single random sequence addition replicate, limiting the search to 50 min per dataset and storing a maximum of 1000 trees per search. We then used these trees to calculate taxon instability indices for each species using the headless version of MESQUITE [33] and pruned, alternatively, the 5% and 10% least stable taxa from each dataset, comparing the effect of the two pruning stringencies (see Additional File 1).

Phylogenetic analyses of the pruned datasets were carried out in PAUP* using 10 random sequence addition replicates to search for most parsimonious trees followed by 100 bootstrap replicates each limited to 30 min (50 h total). Settings for phylogenetic analyses followed those developed in reference [28]. We counted the number of resolved nodes in 50% and 95% majority-rule bootstrap consensus trees as a measure of phylogenetic information. In order to calculate the total percentage of nodes in each tree that were supported, we divided the number of resolved nodes by the total number of nodes in a fully bifurcating tree of all described species in that clade (*N*-2, where *N *is the number of described species in the clade). The numbers of described species in each clade were summed from recent comprehensive checklists and the NCBI taxonomy files [34-41].

### Single gene analyses

We selected a 'heavily studied' subset of the 100 clades, defined as all clades for which at least 20 phylogenetically informative gene clusters were available, to use for an analysis of gene performance. We took the aligned clusters from 2008 that were combined into a supermatrix in the previous analyses and analysed them independently, as single genes. These analyses were carried out identically to the final supermatrix analyses and we scored phylogenetic resolution as the number of resolved nodes in each tree divided by the number of taxa in the tree minus two. We then chose the five largest (in terms of number of taxa sampled) and the five most highly resolved genes for each of these 37 clades and analysed the performance of these markers in order to see if the most heavily studied genes were also among the top-performing.

Alignments and analyses were computationally intensive, requiring several months on 18 fast processors. All automation was implemented in Perl (code available from corresponding author) and analyses were carried out on computers running PhyLIS [42]. We carried out additional analyses at several steps of the automated pipeline to ensure that the automation was working appropriately. We checked that the tree searches were thorough enough to avoid artificially decreasing support and that the rogue taxon pruning did not artificially increase support. We also verified that the automated alignments were performing well (see Additional File 1 for a full description of the error checking analyses).

## Abbreviations

NCBI: National Center for Biotechnology Information; RAG: recombination activating gene.

## Authors' contributions

RCT designed and performed research. RCT and HBS analysed the data and wrote the manuscript.

## Supplementary Material

Additional file 1 **Supplementary results and discussion**. Additional results and discussion pertaining to the phyloinformatic pipeline developed for this study. Click here for file

Additional file 2 **Table S2**. Species sampling and phylogenetic resolution for each of 100 sampled clades. Click here for file

Additional file 3 **Phylogenies for 100 vertebrate clades **A zip file containing the majority rule bootstrap consensus phylogenies for each of the clades that we analysed. These require freely available tree viewing software, such as FigTree or TreeView. Click here for file
