# Supplementary material for: Rapid progress on the vertebrate tree of life
Source: BMC Biol. 2010 Mar 8;8:19. doi: 10.1186/1741-7007-8-19 (PMC2842240; doi:10.1186/1741-7007-8-19)

# Supplementary Results and Discussion

### Dataset characteristics

We excluded data from 18 model taxa which had been sequenced for between ~10,500 sequences (*Canus latrans*) to ~1,700,000 sequences (*Mus musculus*) in GenBank. All but 3 of the model organisms had well-over 100,000 sequences. Sequence filtering resulted in the removal of 17,519 sequences that we deemed unsuitable for phylogenetic analysis based on annotation information, leaving 209,810 sequences for analysis. These sequences were clustered into 3,205 independent single linkage clusters. Yearly sets of the sequences in these clusters were aligned and assembled into 1,192 supermatrices, composed of 10,163 aligned clusters.

Traditional phylogenetic datasets vary along two axes (number of taxa and number of characters). Supermatrix datasets also vary along a third axis, data density or the proportion of missing data. For our datasets, data density was inversely related to the other two axes, with particularly species rich and character rich datasets having decreased density (Fig. 3). In many cases, the most character rich datasets had exceedingly low density, indicating that many of the characters only existed for one or a few taxa in the dataset. Had model-organisms been included in the analysis this pattern would likely have been far stronger. The densest datasets were always small in terms of both taxon and character sampling. The two densest datasets were among the smallest and were both ray-finned fish clades (the Doradidae and the Gymnotiformes), while the least dense were uniformly very large (Fig. 3, additional file 2: Table 2).

### Taxon instability

We calculated a standardized metric of instability for each species and pruned the 5% and 10% least stable taxa. To assess what effect these thresholds had on our results,

we compared the results to each other, as well as to the results from our preliminary analyses where no taxa were pruned. For each of the 100 clades, we plotted the number of nodes resolved in the majority rule consensus tree derived from one threshold versus the same value for the other threshold, as well as the 10% threshold versus the results from the analysis where no taxa were pruned (Fig. 5). Comparing the two thresholds shows that which is used makes little difference, with an average gain in resolution for the 10% threshold over the 5% of 0.04 nodes (ranging from a 16 node gain to a 17 node loss). In contrast, a comparison of the 10% threshold to no pruning shows large differences, with an average gain of 5 nodes per clade (ranging from a 55 node gain to a 7 node loss). These results indicate that unstable taxa have a clear impact on consensus trees, hiding phylogenetic signal and making results overly conservative (see reference [28] for further discussion). However, it appears that the results aren't very sensitive to the particular threshold value employed. This is likely the case because overly-aggressive pruning (i.e, pruning non-rogue taxa in addition to the rogue taxa) will generally only result in a small additional change in overall resolution, because non-rogue taxa tend to be relatively stable across trees and so their removal doesn't effect the support scores for many nodes when these trees are combined into a consensus. We used the results from the less-aggressive 5% threshold analysis throughout the paper.

### Tree searches

Because computational constraints for a dataset of this size required that we employ relatively fast tree search settings, we checked for an effect of tree size on resolution of the consensus trees. If the tree searches were not extensive enough to thoroughly search tree space, we would expect that individual trees from each of the bootstrap replicates employed in our search would be more different from each other than they

would be in a thorough tree search. This is the case because tree searches are less likely to converge on global optima when the tree search is not long enough. Further, this effect would be greater for large clades than for small clades because the number of possible trees grows rapidly with the number of taxa, requiring a much more thorough tree search in order to guarantee that the global optimum is found in each replicate. To check for this effect, we regressed the number of taxa contained in each tree against the resolution of each tree. The data show a very slight, non-significant, downward trend (slope = -0.0003, $r^2 = 0.026$, $P = 0.11$). If our tree searches were not extensive enough, then the effect appears to be mild and, if present, makes our results slightly conservative by decreasing support values.

### Effects of alignment and conflicting data

Resolution decreased slightly from one year to the next for some clades (e.g., Crocodylians in Fig. 2c). This could be due to the addition of data to GenBank with conflicting phylogenetic signal, or could be due to misalignment of sequences. The latter explanation would be an artifact of the informatics pipeline and so is undesirable, while the former is an interesting consequence of variation among gene trees. While it isn't possible to check all the alignments by hand (there are 10,163 independent alignments), we plotted a histogram of year-to-year changes in the phylogenetic resolution for each clade in order to assess the magnitude of the overall phenomenon (Fig. 6). Phylogenetic resolution decreased in only 6% of all year-to-year comparisons, and in only 1% of the comparisons was this decrease large (>5% of the clade's resolution). Our previous work found that our automated alignment and concatenation procedures worked well, but did have low error rates, usually when very divergent or length-heterogeneous sequences passed through our upstream screening [28]. We suspect that the small decreases in resolution here are largely due

to this effect.

## Figures

**Figure 5 - Comparison of alternative strategies for pruning rogue taxa**

The x-axis plots the number of nodes resolved in each tree for the 10% pruning threshold. This is plotted against the same values for the 5% pruning threshold (closed circles) and no pruning of rogue taxa (open circles). The x=y line represents no change in resolution as a result of alternative pruning strategies, points falling below the line indicate an increase in resolution when using the 10% pruning threshold. Comparison of the 5% threshold to no pruning (not shown) is similar to the 10% versus no pruning comparison. Data come from the complete datasets (i.e. 2008 datasets).

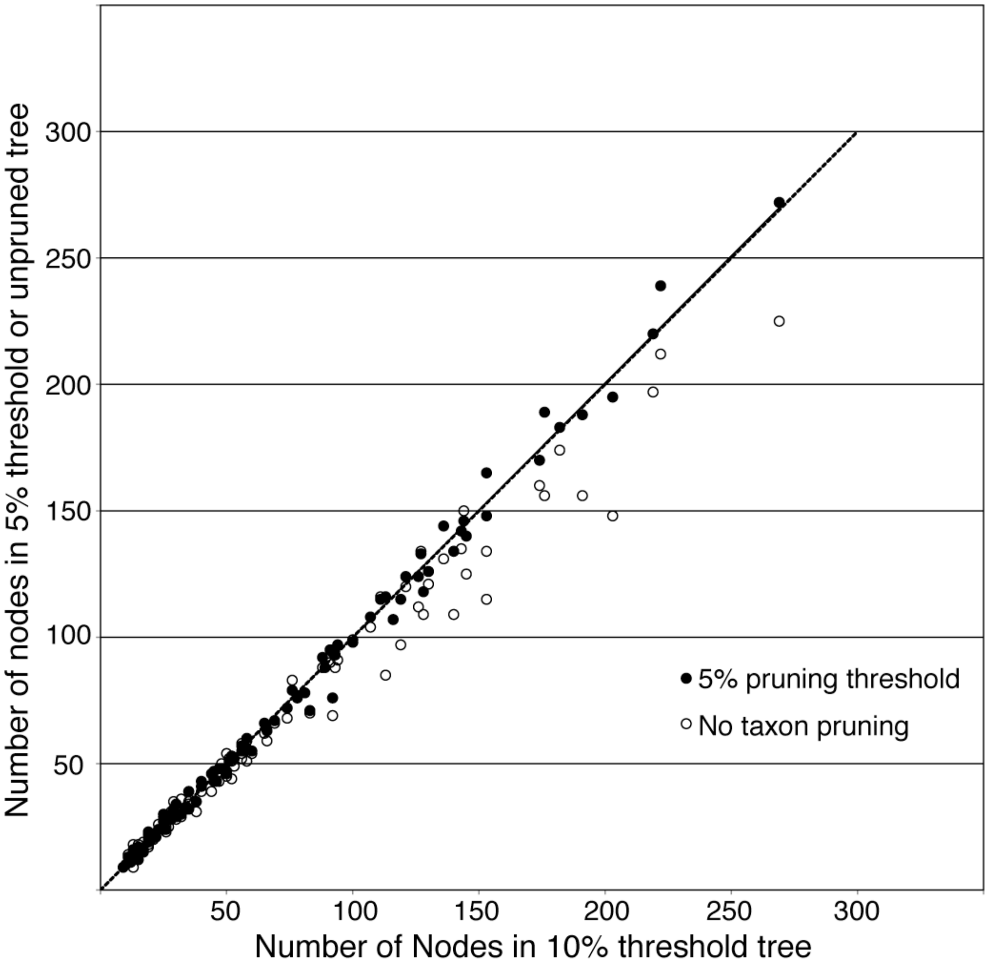

**Figure 6 - Year-to-year changes in resolution among datasets**

The x-axis shows the change in resolution from one year to the next for all year-to-year comparisons (n = 1515). Gray boxes highlight the 6% of cases where resolution decreased slightly (light gray), and 1% of cases where resolution decreased strongly (dark gray).

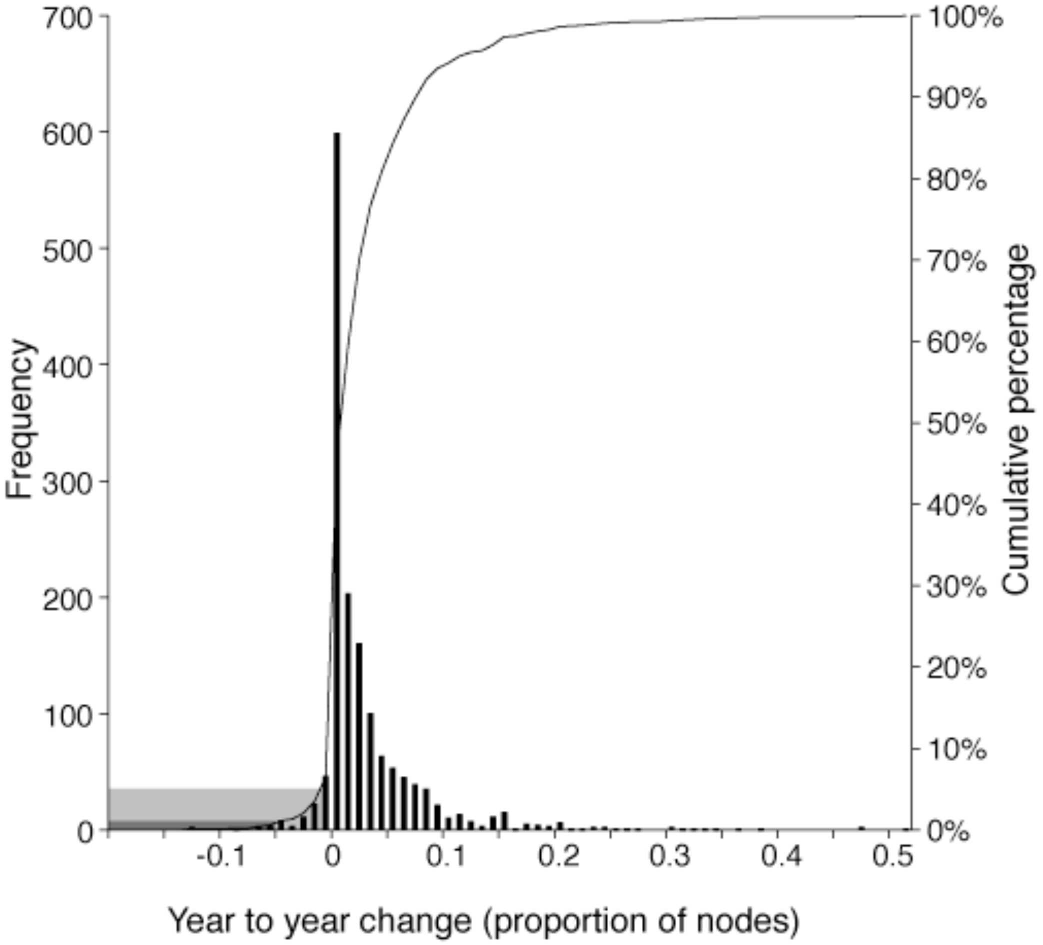

Supplement: Additional file 1 — Supplementary results and discussion. Additional results and discussion pertaining to the phyloinformatic pipeline developed for this study. [file 1741-7007-8-19-S1.PDF]
