# Supplementary material for: Rapid progress on the vertebrate tree of life
Source: BMC Biol. 2010 Mar 8;8:19. doi: 10.1186/1741-7007-8-19 (PMC2842240; doi:10.1186/1741-7007-8-19)
Supplement: Additional file 2 — Table S2. Species sampling and phylogenetic resolution for each of 100 sampled clades. [file 1741-7007-8-19-S2.PDF]

Table 2 - Species sampling and phylogenetic resolution for each of 100 sampled clades

Species diversity is the total number of described species in each clade, assembled from recent comprehensive checklists [34-41]. Proportion of species sampled refers to the number of species included in the analysis, and resolution refers to the number of nodes in a 50% or 95% majority rule bootstrap consensus tree divided by the number of nodes in the fully resolved tree for all described species in that clade.

Clade	Species Diversity	Proportion of Species Sampled	Resolution (50% BP)	Resolution (95% BP)
Acanthuroidei	123	0.66	0.46	0.10
Afrotheria	85	0.42	0.35	0.10
Amphisbaenia	169	0.11	0.08	0.02
Anguilliformes	737	0.18	0.08	0.02
Anguimorpha	122	0.76	0.44	0.06
Anseriformes	162	0.90	0.48	0.16
Aplocheiloidei	560	0.41	0.26	0.09
Apodiformes	104	0.42	0.34	0.11
Atheriniformes	393	0.26	0.11	0.00
Aulopiformes	234	0.14	0.04	0.00
Bagridae	210	0.30	0.11	0.01
Beloniformes	186	0.44	0.31	0.14
Beryciformes	121	0.31	0.13	0.02
Blennioidei	732	0.12	0.04	0.01
Boidae	74	0.64	0.45	0.20
Bucerotiformes	57	0.42	0.31	0.24
Bufonidae	520	0.39	0.24	0.10
Caprimulgiformes	120	0.47	0.30	0.07
Carangoidei	149	0.68	0.32	0.06
Carnivora	283	0.28	0.17	0.04
Centrolenidae	149	0.51	0.38	0.17
Certhiidae	103	0.77	0.43	0.07
Cetacea	90	1.00	0.61	0.28
Chamaeleonidae	130	0.91	0.62	0.19
Characiformes	1495	0.13	0.06	0.02
Chondrichthyes	1158	0.45	0.19	0.04
Ciconiiformes	145	0.54	0.37	0.15
Cirrhitoidea	68	0.66	0.37	0.05
Cisticolidae	111	0.44	0.22	0.03

Clupeiformes	397	0.28	0.14	0.04
Corvoidea	465	0.87	0.52	0.18
Cotingidae	71	0.58	0.35	0.19
Cottoidei	654	0.28	0.14	0.05
Crocodylidae	23	1.00	0.80	0.60
Cryptobranchoidea	55	0.91	0.75	0.50
Cuculiformes	141	0.38	0.22	0.04
Cyprinodontoidei	497	0.61	0.38	0.17
Doradidae	90	0.38	0.28	0.16
Elapidae	300	0.34	0.23	0.10
Falconiformes	306	0.70	0.54	0.23
Galliformes	284	0.77	0.48	0.17
Gekkota	1060	0.40	0.21	0.05
Gobiidae	1875	0.13	0.05	0.01
Gruiformes	191	0.40	0.24	0.10
Gymnophiona	176	0.20	0.17	0.05
Gymnotiformes	155	0.09	0.06	0.04
Hylidae	860	0.46	0.32	0.16
Hystricognathi	244	0.69	0.48	0.26
Ictaluridae	45	0.91	0.64	0.36
Iguaninae	34	0.94	0.65	0.16
Lacertoidea	268	0.77	0.43	0.17
Lagomorpha	83	0.90	0.58	0.15
Laridae	103	0.99	0.57	0.22
lemuriformes	60	1.00	0.84	0.44
Lutjanidae	103	0.54	0.28	0.03
Mantellidae	174	0.85	0.42	0.15
Mesobatrachia	187	0.49	0.41	0.23
Metatheria	315	0.77	0.47	0.17
Microhyloidea	567	0.29	0.17	0.07
Muridae	683	0.52	0.29	0.09
Muscicapidae	275	0.38	0.16	0.05
Myctophiformes	241	0.33	0.13	0.03
Nectariniidae	131	0.30	0.09	0.00
Nesomyidae	53	0.57	0.38	0.16
Osteoglossiformes	214	0.34	0.24	0.16
Palaeognathae	73	0.45	0.30	0.09
Paracanthopterygii	1207	0.06	0.02	0.00
Pelicaniformes	67	0.76	0.64	0.39
Percidae	195	0.99	0.69	0.40
Perissodactyla	20	1.00	0.76	0.47
Petromyzontiformes	41	0.61	0.32	0.08
Phyllostomidae	155	0.94	0.71	0.30
Plethodontidae	384	0.71	0.48	0.19
Polychrotinae	450	0.45	0.33	0.16
Pomacanthidae	74	0.58	0.42	0.17

Pomacentridae	321	0.46	0.29	0.13
Procellariiformes	127	0.99	0.74	0.30
Psittaciformes	368	0.61	0.32	0.12
Pycnonotidae	130	0.78	0.50	0.09
Rhacophoridae	296	0.30	0.18	0.07
Rhinolophidae	145	0.46	0.23	0.02
Sciaenidae	270	0.25	0.08	0.02
Sciuridae	276	0.63	0.45	0.17
Scorpaenoidei	442	0.38	0.22	0.10
Serranidae	449	0.44	0.24	0.07
Sparidae	112	0.58	0.43	0.15
Stenopterygii	333	0.14	0.05	0.01
Strigiformes	215	0.47	0.31	0.09
Stromateoidei	65	0.57	0.48	0.16
Sylviidae	291	0.73	0.44	0.15
Testudines	321	0.77	0.59	0.26
Tetraodontiformes	353	0.33	0.21	0.08
Thamnophilidae	212	0.57	0.37	0.20
Trochilidae	339	0.57	0.42	0.17
Typhlopoidea	305	0.12	0.07	0.01
Vespertilionidae	364	0.62	0.39	0.14
Viperidae	228	0.92	0.52	0.23
Xenarthra	27	0.67	0.58	0.33
Xenodontinae	300	0.20	0.09	0.03
Zoarcoidei	312	0.21	0.07	0.00
